# Enhancing the Internet of Medical Things (IoMT) Security with Meta-Learning: A Performance-Driven Approach for Ensemble Intrusion Detection Systems

**DOI:** 10.3390/s24113519

**Published:** 2024-05-30

**Authors:** Mousa Alalhareth, Sung-Chul Hong

**Affiliations:** 1 Department of Information Systems, College of Computer Science and Information System, Najran University, Najran 61441, Saudi Arabia; 2 Department of Computer and Information Sciences, Towson University, Towson, MD 21204, USA

**Keywords:** IoMT, IDS, meta-learning, ensemble models, cybersecurity

## Abstract

This paper investigates the application of ensemble learning techniques, specifically meta-learning, in intrusion detection systems (IDS) for the Internet of Medical Things (IoMT). It underscores the existing challenges posed by the heterogeneous and dynamic nature of IoMT environments, which necessitate adaptive, robust security solutions. By harnessing meta-learning alongside various ensemble strategies such as stacking and bagging, the paper aims to refine IDS mechanisms to effectively counter evolving cyber threats. The study proposes a performance-driven weighted meta-learning technique for dynamic assignment of voting weights to classifiers based on accuracy, loss, and confidence levels. This approach significantly enhances the intrusion detection capabilities for the IoMT by dynamically optimizing ensemble IDS models. Extensive experiments demonstrate the proposed model’s superior performance in terms of accuracy, detection rate, F1 score, and false positive rate compared to existing models, particularly when analyzing various sizes of input features. The findings highlight the potential of integrating meta-learning in ensemble-based IDS to enhance the security and integrity of IoMT networks, suggesting avenues for future research to further advance IDS performance in protecting sensitive medical data and IoT infrastructures.

## 1. Introduction

The healthcare sector has undergone a significant transformation with the rise of the Internet of Things (IoT) and the emergence of the Internet of Medical Things (IoMT), aimed at improving patient quality of life [1]. In this context, the application of ensemble learning in intrusion detection systems (IDS) for IoT and IoMT networks presents a promising approach to enhance cybersecurity measures. Researchers have explored various ensemble techniques, such as stacking, bagging, and boosting, to optimize the combination of diverse base models for improved predictive accuracy and robustness in detecting anomalies within IoT and IoMT environments [2,3]. The integration of ensemble learning in IDS for IoT and IoMT networks addresses the critical need for reliable security solutions to protect sensitive medical data and ensure the integrity of healthcare systems amidst the proliferation of connected devices and services [4,5]. The use of ensemble learning in IDS for IoT and IoMT networks provides a sophisticated approach to combat cyber threats and enhance network security. By utilizing meta-learning and ensemble techniques, researchers can develop adaptive intrusion detection mechanisms that dynamically adjust decision fusion strategies based on evolving attack patterns and network conditions [6,7]. The combination of machine learning algorithms, deep learning models, and ensemble methods in IDS for IoMT networks not only improves anomaly detection capabilities but also contributes to the creation of lightweight and efficient security solutions tailored to the unique challenges posed by interconnected medical devices and IoT ecosystems [8,9]. As the field of IoT security evolves, the application of ensemble learning in IDS for IoT and IoMT networks holds significant potential in strengthening healthcare systems against cyber threats and ensuring the confidentiality and integrity of patient data [10,11].

Ensemble-based intrusion detection systems (IDSs) for the Internet of Medical Things (IoMT) and the Internet of Things (IoT) face several challenges that impact their effectiveness and reliability. One significant challenge is the inherent heterogeneity and resource constraints of devices within IoMT and IoT networks, leading to difficulties in standardizing security measures and ensuring consistent protection across all connected devices [12,13,14]. Moreover, the dynamic and evolving nature of cyber threats in healthcare environments poses a challenge for ensemble-based IDSs, as they must continuously adapt to new attack vectors and vulnerabilities to maintain robust security measures [15,16]. The complexity of data fusion and analysis in IoMT and IoT environments further complicates the detection of anomalies and intrusions, requiring sophisticated ensemble learning techniques to effectively identify malicious activities while minimizing false positives [17,18]. Additionally, the privacy and confidentiality of sensitive medical data present a critical challenge for ensemble-based IDSs in IoMT and IoT networks, as unauthorized access or data breaches can have severe consequences for patient safety and trust in healthcare systems [18,19]. The scalability and interoperability of security mechanisms across diverse IoMT and IoT devices also pose challenges for ensemble-based IDSs, as they must ensure seamless integration and communication while maintaining robust security protocols [20,21]. Furthermore, the rapid proliferation of connected devices and the increasing complexity of IoMT and IoT ecosystems introduce challenges in managing and securing a vast network of interconnected devices, requiring ensemble-based IDSs to adapt to the scale and diversity of these environments to effectively mitigate security risks [2,22].

To address the challenges, leveraging meta-learning techniques can play a crucial role in optimizing the combination of diverse base models within ensemble systems, enhancing the adaptability and performance of IDSs in detecting anomalies and cyber threats [23,24]. By utilizing meta-learning, IDSs can dynamically adjust decision fusion strategies based on evolving attack patterns and network conditions, improving the robustness and reliability of intrusion detection mechanisms in the IoMT environment [25,26]. Moreover, meta-learning can aid in addressing the interpretability challenges of ensemble models, providing insights into how predictions are generated and enhancing trust in the decision-making process of IDSs [27,28]. Furthermore, meta-learning can help overcome the scalability challenges of ensemble-based IDSs in IoMT and IoT networks by optimizing computational resources and improving efficiency in handling large datasets and real-time applications [29,30]. By incorporating meta-learning techniques, IDSs can enhance their generalization capabilities across diverse datasets and domains, ensuring consistent performance and adaptability to varying network conditions and security threats [31,32]. Additionally, meta-learning can contribute to the development of robust and efficient security measures in the IoMT, addressing privacy concerns, data integrity issues, and the interoperability of security mechanisms across connected devices [33,34]. Overall, the integration of meta-learning in ensemble-based IDSs for the IoMT holds significant promise in advancing cybersecurity measures and ensuring the integrity and confidentiality of sensitive data in connected environments. 

Ensemble learning based on meta-learning encounters several challenges that affect its effectiveness and applicability in various domains. One significant challenge is the complexity of integrating multiple base models and meta-learners, necessitating careful consideration of model selection, hyperparameter tuning, and feature engineering to optimize the ensemble’s performance [35,36]. The interpretability of meta-ensemble models poses another challenge, as the decision-making process may become opaque due to the intricate interactions between base models and meta-learners, hindering the understanding of how predictions are generated and affecting trust in the model’s outcomes [37,38]. Moreover, the scalability of meta-learning-based ensembles presents a challenge when dealing with large datasets or real-time applications, as the computational resources required for training and inference can be substantial, impacting the model’s efficiency and practicality [39,40]. Furthermore, the generalization of meta-learning-based ensembles across diverse datasets and domains poses a challenge, as the performance of the ensemble may vary significantly depending on the characteristics of the data and the task at hand [41,42]. The robustness of meta-ensemble models to adversarial attacks and noisy data is another critical challenge, as the model’s decision-making process may be vulnerable to perturbations or misleading inputs, leading to compromised performance and reliability [43,44]. Additionally, the lack of standardized evaluation metrics and benchmark datasets for meta-learning-based ensembles hinders the comparison of different approaches and the reproducibility of results, posing challenges in assessing the model’s performance and generalizability [45,46]. Addressing these challenges is crucial to advancing the field of meta-learning-based ensembles and unlocking their full potential in various applications and domains.

Architectural diversity and decision aggregation in ensemble deep learning intrusion detection system (IDS) models for the IoMT present several challenges and considerations. Ensemble deep learning models combine multiple base models to enhance predictive accuracy and reliability through decision fusion strategies [47]. These models encompass various ensemble techniques like bagging, boosting, stacking, and negative correlation-based models, each with unique characteristics [48]. The aggregation of diverse architectures in ensemble models, including homogeneous/heterogeneous ensembles, requires careful consideration to leverage the strengths of each model effectively [49]. In the context of IDS, ensemble learning plays a crucial role in improving detection accuracy by combining the outputs of multiple classifiers [50]. However, the interpretability of individual ensemble members may be lost during the aggregation process, highlighting a trade-off between accuracy and explainability [51]. Moreover, the selection of appropriate ensemble learning models is essential to address the specific requirements of IDS, considering factors such as feature selection and model performance [52]. Decision aggregation in ensemble deep learning IDS models involves combining decisions from multiple submodels to enhance prediction accuracy and overall performance [53]. This process requires careful consideration of how decisions are fused to ensure optimal results. Additionally, the use of ensemble learning in IDS frameworks, such as the all predict wisest decides (APWD) model, demonstrates the importance of selecting the most appropriate model for each class to improve intrusion detection capabilities [54]. In conclusion, addressing the issues related to architectural diversity and decision aggregation in ensemble deep learning IDS models requires a comprehensive understanding of ensemble techniques, careful selection of models, and thoughtful decision fusion strategies. By leveraging the strengths of diverse architectures and optimizing the aggregation process, the accuracy and reliability of IDS systems can be improved.

To address the architectural diversity in ensemble deep learning IDS models, we implement a meta-learning approach. Meta-learning involves training a meta-model that learns how to best combine the outputs of diverse base models based on the characteristics of each model and the specific task at hand. By utilizing meta-learning, the ensemble system can adaptively adjust the decision aggregation process to optimize performance, considering the strengths and weaknesses of each individual model. Meta-learning has been successfully applied in various domains to improve the performance of ensemble models by dynamically adjusting the combination of base models based on the input data and the current task requirements. In the context of IDS for the IoMT, meta-learning can help in effectively leveraging the architectural diversity present in ensemble models by learning the optimal way to aggregate decisions from different models for intrusion detection tasks. By incorporating a meta-learning component into the ensemble deep learning IDS model, we can enhance the adaptability and performance of the system, addressing the challenges posed by architectural diversity. This solution allows the ensemble system to dynamically adjust its decision aggregation strategy based on the input data and the characteristics of the base models, leading to improved detection accuracy and reliability in intrusion detection systems for the IoMT.

To this end, the contribution of this paper is three-fold as follows:

- A performance-driven weighted meta-learning technique was developed to dynamically assign voting weights to each classifier in the ensemble based on accuracy, loss, and level of confidence of prediction.
- A meta-learning-based ensemble model was developed to detect intrusion attacks on the IoMT by incorporating the technique into (i) the ensemble IDS model.
- An extensive experimental evaluation was conducted by investigating the performance of the mode over various sizes of input features and comparing the performance with existing models.

The rest of the paper is organized as follows: In Section 2, related works were explored. Section 3 describes the methodology and proposed techniques. Section 4 presents and discusses the experimental results and comparison with related models. The paper ends with a conclusion section.

## 2. Related Works

Meta-learning has been recognized as a powerful technique for enhancing the performance of ensemble models in various domains. By training a meta-model to effectively combine outputs from different base models, meta-learning allows the ensemble system to dynamically adjust its decision aggregation process based on individual model characteristics and specific task requirements [55]. Research in drug discovery and sentiment analysis has illustrated the efficacy of meta-ensemble deep learning methods in improving predictive accuracy by leveraging the interpretability of meta-learning to combine individual models efficiently [36]. Moreover, meta-learning-based ensemble models have demonstrated superior performance in tasks such as barrier layer thickness estimation and microRNA prediction, surpassing individual models in terms of accuracy and spatial distribution [56]. The application of meta-learning in ensemble models for tasks like time series forecasting, energy consumption prediction, and network traffic classification has shown promising outcomes in enhancing model generalizability and robustness [57]. The stacking ensemble approach, which consolidates predictions from multiple machine learning models into a single meta-learner model, has proven particularly effective in accelerating predictions and enhancing overall performance [58]. By utilizing meta-learning techniques in ensemble models, researchers can achieve improved performance by amalgamating predictions from multiple models, ultimately leading to more robust and accurate outcomes across a wide array of applications [59].

The utilization of meta-learning in ensemble models for intrusion detection systems (IDS) offers a promising approach to enhance the accuracy and efficiency of anomaly detection. By training a meta-model to dynamically adjust the decision aggregation process based on the characteristics of individual base models and specific intrusion detection tasks, meta-learning enables the ensemble system to optimize performance by effectively combining diverse architectural models [60]. Research in the field of IDS has shown that meta-learning-based ensemble models can adaptively adjust their decision fusion strategies, leading to improved detection capabilities and robustness against network attacks [61]. Furthermore, a systematic literature review conducted on intrusion detection systems using ensemble learning approaches highlights the effectiveness of meta-learning in protecting network infrastructures from intruders and suspicious activities, showcasing a significant improvement over traditional methods [33]. The application of meta-learning in ensemble IDS models not only enhances detection accuracy but also contributes to the interpretability and adaptability of the system. By leveraging meta-ensemble approaches, researchers can address the challenges posed by architectural diversity in IDS models, leading to more reliable and efficient intrusion detection mechanisms [60]. The integration of meta-learning techniques in ensemble models for IDS can facilitate the identification of complex network intrusions and improve the overall security posture of systems by dynamically adjusting decision aggregation strategies based on evolving threats and attack patterns [61]. Overall, the use of meta-learning in ensemble IDS models represents a cutting-edge approach that holds great potential for advancing the field of intrusion detection and cybersecurity.

Meta-learning in ensemble models involves various methods to optimize the combination of diverse base models for improved predictive accuracy and robustness. Techniques such as bagging, boosting, and stacking are commonly employed in ensemble learning to enhance model performance by leveraging the strengths of individual models and mitigating their weaknesses [62]. Meta-ensemble methods, which involve combining multiple ensemble techniques, offer a comprehensive approach to adaptively adjust decision fusion strategies based on the characteristics of each base model, leading to superior predictive capabilities in tasks such as sentiment analysis and disease detection [36,63]. Additionally, the use of feature importance permutation methods and hyperparameter optimization in meta-learning on ensemble models allows for the evaluation of predictor contributions and the fine-tuning of model parameters to achieve optimal performance [43,63]. Furthermore, meta-learning techniques in ensemble models facilitate the development of sophisticated approaches like stacking, where predictions from multiple base models are combined into a meta-learner model to improve overall accuracy and generalizability [57]. By exploring different ensemble methods such as bagging, boosting, and stacking, researchers can effectively address the challenges of architectural diversity and decision aggregation in ensemble deep learning IDS models, leading to more reliable and efficient intrusion detection systems [64]. The integration of meta-learning in ensemble models not only enhances model interpretability and adaptability but also contributes to the advancement of various domains, including healthcare, finance, and environmental science, by providing robust and accurate predictive models [65,66,67].

## 3. The Methodology

**Figure 1:**
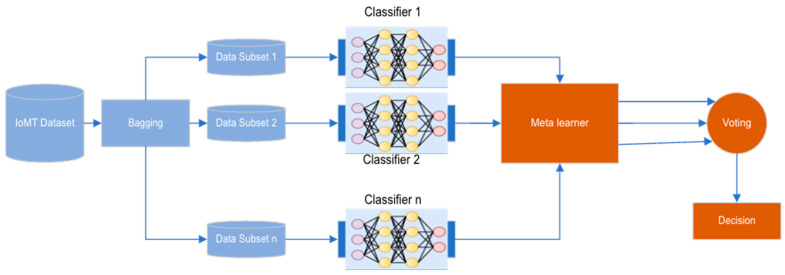
The structure of proposed IDS model.

In the evolving landscape of the Internet of Medical Things (IoMT), safeguarding against cyber threats necessitates sophisticated intrusion detection systems (IDS). Our research introduces an innovative ensemble architecture that leverages a suite of advanced deep learning classifiers, including convolutional neural networks (CNNs), recurrent neural networks (RNNs), and autoencoders, each trained on a dataset rich in IoMT-specific intrusion scenarios. This architecture is not only designed to identify the complex patterns characteristic of IoMT data but also to adapt and respond to emergent cyber threats dynamically. Central to our approach is the development of a meta-learner, a strategic component engineered to intelligently orchestrate the various classifiers within the ensemble. By continuously evaluating and optimizing the ensemble’s structure through real-time performance metrics, the meta-learner enhances the IDS by selectively activating and weighting the most effective models. This allows our system to focus its computational prowess where it is most needed, ensuring high adaptability, accuracy, and efficiency in threat detection within IoMT environments. Figure 1 shows the structure of the proposed ensemble meta-learning IDS model. It consists of three main components: the data component, where the original dataset is split into n of subsets using the bagging approach; the ensemble’s base classifiers’ component, where each classifier makes an individual decision; and the meta-learner component that receives the individual decisions and reweight them before making the final decision based on voting. 

### 3.1. A Performance-Driven Meta-Learner Weighting Technique 

Our ensemble architecture integrates a selection of deep learning classifiers ($D_{j}$, where j=1,2,…,m), including convolutional neural networks (CNNs), recurrent neural networks (RNNs), and autoencoders. These classifiers are trained on a dataset encompassing a diverse range of intrusions pertinent to the IoMT, enabling them to capture and learn from the complex patterns inherent in such data.

The development of a meta-learner for orchestrating deep learning classifiers within an ensemble framework represents a pivotal component of our methodology, designed to bolster the IDS for the IoMT. This meta-learner is engineered to intelligently navigate the complexities of IoMT data, leveraging the nuanced capabilities of deep learning models to detect and respond to cyber threats. At its core, the meta-learner dynamically assesses and optimizes the composition and configuration of the ensemble. It extracts and analyzes meta-features from the outputs of each deep learning classifier based on classification accuracy ($A_{j}$), loss metrics ($L_{j}$), and prediction confidence levels ($C_{j}$). These meta-features serve as a basis for understanding the current efficacy and relevance of each classifier.

The meta-learner applies a strategic optimization process to determine the optimal selection $\left(S(D_{j})\right)$ and weighting $\left(W(D_{j})\right)$ of deep learning classifiers within the ensemble. The selection function (S) identifies which classifiers are most suited to address the current threat dynamics, ensuring that only the most effective models are active at any given time. Concurrently, the weighting function (W) allocates relative importance to each selected classifier, adjusting their influence on the ensemble’s overall decision-making process based on real-time performance metrics. This dual-function approach enables the ensemble to adaptively recalibrate its strategy, focusing computational resources on classifiers that offer the greatest contribution to detecting and mitigating intrusions. The meta-learner’s ability to continuously refine the ensemble’s composition and weights in response to emerging threats and changing IoMT environments underscores its innovative role in enhancing the IDS’s adaptability, accuracy, and efficiency.

#### 3.1.1. Meta-Feature Extraction

Let $D_{j}$ be a deep learning classifier within the ensemble, where j=1,2,...,m. Each classifier $D_{j}$ is associated with a set of meta-features *F_j_* = {*A_j_*, *L_j_*, *C_j_*}, where

- $A_{j}$ is the accuracy of classifier $D_{j}$,
- $L_{j}$ is the loss metric for classifier $D_{j}$,
- $C_{j}$ is the confidence level of the predictions made by $D_{j}$.

These meta-features are extracted from the outputs of each classifier and serve as the inputs to the meta-learner.

#### 3.1.2. Meta-Learner Optimization Functions

The meta-learner employs two primary functions: the selection function $S(D_{j})$ and the weighting function $W(D_{j})$.

**Selection Function** (S): This function determines whether a classifier $D_{j}$ should be included in the ensemble. It can be represented as a binary decision function where $S(D_{j})\in \{0,1\}$, with 1 indicating inclusion and 0 indicating exclusion. The decision is based on a threshold mechanism or criteria defined on the meta-features $F_{j}$.

**Weighting Function** (W): This function assigns a weight to each selected classifier, indicating its relative importance within the ensemble. The weights are normalized to ensure that.

(1) $$\sum_{j=1}^{m}W\left(D_{j}\right)=1$$

where $W(D_{j})>0$ for all selected classifiers. The weighting function optimizes the ensemble’s overall performance metric, which could be a combination of accuracy, precision, recall, or any other relevant metric to the IDS objectives.

#### 3.1.3. Ensemble Performance Optimization

The overall performance of the ensemble, $P_{ensemble}$, is a function of the weighted contributions of all selected classifiers. It can be represented as the following:

(2) $$P_{ensemble}=\sum_{j=1}^{m}W\left(D_{j}\right).P(D_{j})$$

where $P(D_{j})$ is the performance metric of classifier $D_{j}$, which could be $A_{j}$, or a composite metric derived from the meta-features $F_{j}$.

#### 3.1.4. Objective Function

The meta-learner’s goal is to maximize $P_{ensemble}$ subject to computational constraints and the dynamic nature of the IoMT threat landscape. This can be formulated as an optimization problem:

(3) $$\underset{\mathrm{sw}}{\max}P_{ensemble}$$

subject to

(4) $$\sum_{j=1}^{m}\Omega_{j}\left(D_{j}\right)\leq \Omega_{m}$$

where $\Omega_{j}\left(D_{j}\right)$ represents the computational cost of including classifier $D_{j}$ in the ensemble, and $\Omega_{m}$ is the maximum allowable computational budget for the ensemble.

This mathematical framework underpins the adaptive, real-time decision-making capabilities of the meta-learner, enabling the dynamic selection and weighting of deep learning classifiers to optimize the IDS’s performance in the face of evolving IoMT security challenges. Algorithm 1 shows the pseudocode for the proposed meta-learner optimization approach. (The Pseudocode for PMWT Technique. Note: The aggregation of predictions in Evaluate_Ensemble could use techniques like weighted voting or averaging, where the weight of each classifier’s vote or prediction is proportional to its updated weight Wi).
**Algorithm 1**. Meta-Learner_OptimizationInput: Ensemble of deep learning classifiers {C1, C2,…, C7}, Training data D Output: Optimized weights for each classifier in the ensemble1: Initialize weights Wi for each classifier Ci in the ensemble, i = 1 to 7, such that sum (Wi) = 1 2: For each training epoch or until performance converges do 3: For each classifier Ci in the ensemble do 4: Extract meta-features: Accuracy Ai, Loss Li, Confidence Level ConfLi from Ci using D 5:  Calculate performance score PSi for Ci using Ai, Li, ConfLi 6:  Update weight Wi for Ci based on PSi 7:  End For 8:  Evaluate ensemble performance on validation set using updated weights 9:  If ensemble performance has converged or improved minimally then 10:    Break from the loop 11:  End If 12: End For Procedure Calculate_Performance_Score(Accuracy Ai, Loss Li, Confidence Level ConfLi) 1:  Define a performance function F that considers Ai, Li, ConfLi 2:  Return performance score PSi = F(Ai, Li, ConfLi) Procedure Update_Weight(Performance Score PSi) 1:  Define a weighting strategy that adjusts Wi based on PSi 2:  Update Wi according to the defined strategy 3:  Normalize all weights Wi so that sum(Wi) = 1 Procedure Evaluate_Ensemble(Validation Data V) 1:  For each data point in V do 2:  Aggregate predictions from all classifiers using their weights Wi 3:  End For 4:  Calculate and return the overall performance of the ensemble on V

### 3.2. Training of Dynamic Ensemble-Based IDS for IoMT

In the development of our ensemble-based intrusion detection system (IDS) for the Internet of Medical Things (IoMT), a pivotal step involves the training of the ensemble model, which is composed of seven deep learning classifiers. To effectively harness the collective strength of these classifiers, we employ the bagging technique—a bootstrap aggregating method that introduces diversity and robustness into the model training process. Specifically, the original training dataset, which is meticulously curated to encompass a broad spectrum of IoMT-specific intrusion scenarios and benign activities, is randomly split into seven smaller subsets using bagging. Each subset is then used to train one of the seven classifiers independently. For the autoencoder’s training, an unsupervised approach was used based on a subset of data that contains only one type of data (either normal or attack). We used two autoencoders (one model for each type). The other five classifiers were trained using the supervised approach, where both normal and attack data were included in the training subsets. Out of the remaining five classifiers, CNN was used to train two LSTM to train two classifiers. This approach not only enhances the generalization ability of individual classifiers by exposing them to varied slices of the data but also mitigates the risk of overfitting, as each classifier learns from a slightly different perspective of the data. The bagging technique, by design, is particularly well-suited for the IoMT environment, where the data can be highly imbalanced and diverse due to the myriad devices and interaction patterns present.

Upon training, each of the seven classifiers develops unique expertise in detecting specific types of intrusions or anomalies within the IoMT network, contributing to a comprehensive coverage of the threat landscape. The ensemble model then aggregates the predictions of these classifiers to make a final decision, leveraging their collective intelligence. The aggregation is overseen by the meta-learner, which dynamically adjusts the weight assigned to each classifier’s vote based on its performance and relevance to the current data stream, ensuring that the most competent classifiers have a proportional influence on the ensemble’s outcome. This adaptive weighting mechanism is crucial for maintaining the ensemble’s effectiveness over time, allowing it to respond adeptly to evolving threats and to the introduction of new IoMT devices and technologies. Through this sophisticated training and aggregation process, the ensemble model achieves a high degree of accuracy and robustness in intrusion detection, embodying a potent defense mechanism against the complex and dynamic cyber threats faced by the IoMT ecosystem.

Although the proposed model can identify cyber threats in IoMT environments, it is important to consider the potential risk of overfitting. Overfitting arises when a model acquires the ability to achieve outstanding performance on the training data, yet it struggles to apply this knowledge to unfamiliar data. This problem holds significant importance in IDS for the IoMT, considering the varied and ever-changing characteristics of cyber threats. The model may develop a high level of specialization in detecting familiar attack patterns found in the training data, which can make it challenging to identify novel or slightly altered attacks. Furthermore, the dynamic weighting mechanism employed in our meta-learning approach has the potential to excessively optimize the model for specific profiles encountered during training, thereby diminishing its capacity to generalize to unfamiliar data distributions. The intricate nature of employing multiple deep learning classifiers amplifies the likelihood of substantial variability, thereby diminishing the stability of the model when confronted with novel data. Particular vulnerabilities encompass zero-day attacks, adversarial attacks, and ever-changing attack strategies. In order to address the issue of overfitting, we employ regularization methods such as dropout, L2 regularization, and cross-validation as part of the training procedure. These techniques aid in preventing the model from becoming excessively intricate and reliant on particular data points. In addition, the model utilizes continuous learning mechanisms and diverse data augmentation techniques to ensure it can adapt to new attack patterns and maintain strong performance. The proposed ensemble IDS model aims to ensure effective intrusion detection in the dynamic IoMT environment by addressing risks and implementing strategies that maintain high performance and adaptability. 

### 3.3. Description of the Dataset

In our research, we utilized the WUSTL-EHMS-2020 dataset, which includes both parameters of network flow and biometric data of patients. This dataset originates from a testbed for an enhanced healthcare monitoring system (EHMS), which operates in real time. The testbed’s architecture is composed of four key components: medical monitoring sensors, a gateway for data transmission, network infrastructure, and a visualization and control unit. Data collection begins with medical sensors attached to patients, progresses through the gateway, and culminates at a server designated for data visualization, employing routing and switching mechanisms for data transfer. The EHMS testbed aims to gather both network flow metrics and patient biometric data within a system built on six essential elements: a multisensor board, a central control hub or gateway, a data server, an IDS, a simulated attacker, and a specialized network.

The PM4100 Six Pe Multi-Sensor Board, sourced from Medical Expo, features four sensors that track vital patient indicators such as ECG, SpO2, body temperature, and blood pressure. These sensors send data to a gateway laptop via USB, which then displays the information through a GUI and forwards it to a server for further processing. The server, running on Ubuntu, collects and analyzes the data to support medical decision-making. The network includes an Ethernet switch connecting the server, IDS, and an attack-simulating computer, with a router managing dynamic IP allocation. The IDS employs Argus-v3.0.8.2 software to collect data on network flow and biometrics, analyzing traffic packets for security. A computer running Kali Linux simulates attacks like data spoofing or altering patient data in transit, representing potential security threats in healthcare monitoring systems.

This setup reflects a realistic IoMT environment, where sensors on patients gather essential health metrics and transmit this data to a gateway. The gateway acts as an intermediary, processing and sending sensor data to the server through the network infrastructure efficiently. The system’s network design features various devices such as switches, routers, and firewalls to facilitate data transmission from the gateway to the server. The server, which handles control and visualization, presents the data in a format that healthcare professionals can easily access and interpret, enabling them to monitor patient health in real time and make well-informed medical decisions based on the visualized data.

The development and evaluation of the suggested model were carried out using a range of software and utilities, including Python-v3.12.2, Skfeature-v1.1.2, TensorFlow-v 2.15, Keras-v2, Scikit Learn-v1.3, and NumPy-v1.26.0. Furthermore, the arrangement of data instances, the implementation of algorithms, and the analysis of findings were conducted on a machine powered by an Intel(R) Core(TM) i7-4790 CPU @ 3.60 GHZ with 16 GB of RAM. This research gauged the efficacy of the proposed model using accuracy as the primary metric for performance. Additional measures of the IDS model’s estimation errors were determined by examining its false positive rate (*FPR*), detection rate (*DR*), and f-score (*F*1), as illustrated in the equations that follow:

(5) $$ACC=\frac{TP+TN}{TP+TN+FP+FN}$$

(6) $$FPR=\frac{FP}{TN+FP}$$

(7) $$DR=\frac{TP}{TP+FN}$$

(8) $$F1=\frac{TP}{TP+0.5*(FP+FN)}$$

where *TP*, *TN*, *FP*, and *FN* denote true positive, true negative, false positive, and false negative, respectively.

## 4. Results and Discussion

In this study, the WUSTL-EHMS-2020 dataset was employed. It comprises network flow parameters and patients’ biometric data of. The dataset is derived from a testbed for an EHMS that functions in real time. The architecture of the testbed consists of four essential components: medical monitoring sensors, a data transmission gateway, network infrastructure, and a visualization and control unit. The dataset is carefully selected to include a wide range of intrusion scenarios specific to the IoMT and normal activities. The dataset includes many records, which guarantees a varied and representative sample for both training and testing. More precisely, the dataset is partitioned into two subsets: a training subset and a testing subset, with a ratio of 80:20. The training set is utilized for model development, while the testing set is exclusively reserved for performance evaluation. To enhance the reliability and reduce the risk of overfitting, we utilized cross-validation as part of the training procedure. The initial training dataset was divided into multiple subsets using the bagging technique, leading to the creation of several smaller datasets. These smaller datasets were then utilized to train individual classifiers within the ensemble. This technique enhanced the generalization ability of the classifiers by exposing them to diverse slices of the data during the model training process, thereby introducing diversity and robustness.

**Figure 2:**
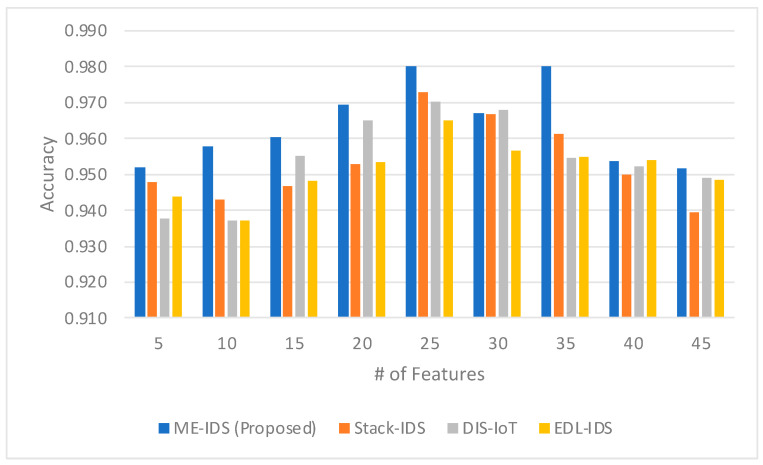
The comparison between the accuracy obtained by the proposed model and the related models.

Figure 2 shows a comparative analysis of accuracy across four different intrusion detection system models with a varying number of features. The proposed model, ME-IDS, exhibits higher accuracy in most instances as the number of features increases, peaking at an accuracy of 0.980 with 25 and 35 features. This model demonstrates a consistent outperformance compared to the other models, particularly noticeable at the 20-feature mark, where it surpasses the nearest competitor, DIS-IoT [68], by 0.005 and exhibits a more significant lead over Stack-IDS [69] and EDL-IDS [70], which stand at 0.953. Notably, ME-IDS maintains a robust accuracy level as the feature count increases, showing only a slight decrease when the number of features is raised from 35 to 45. In contrast, Stack-IDS shows a marked decrease in performance as the number of features grows, with a noticeable dip to 0.939 at the 45-feature mark. DIS-IoT and EDL-IDS show less fluctuation in accuracy as the number of features varies, but neither matches the peak accuracy of ME-IDS. Overall, the proposed ME-IDS model demonstrates a strong performance, particularly in scenarios with an intermediate number of features (20 to 35), suggesting an optimal balance between feature set complexity and model accuracy.

The comparison of accuracy in Figure 2 indicates that the ME-IDS model, enhanced by the integration of a meta-learner for model selection and weighting, along with a self-optimizing ensemble architecture, consistently outperforms the other models across various feature set sizes. The peak accuracy of 0.980 at both 25 and 35 features underscores the efficacy of the meta-learner in dynamically tuning the ensemble. It suggests that the meta-learner is adept at selecting and weighting the most predictive features, thus optimizing the model’s performance. The slight decline in accuracy observed when the feature set expands beyond 35 could imply that there’s an optimal range within which the meta-learner efficiently manages the feature space complexity before it begins to encounter diminishing returns. This effect illustrates the self-optimizing characteristic of the ensemble architecture, which adapts to the changing effectiveness of its constituent models as the feature space evolves. The contrast with other models, such as Stack-IDS, DIS-IoT, and EDL-IDS, which either show more significant performance drops or less pronounced peaks, further highlights the contribution of the meta-learner’s intelligent feature management and the robust adaptability of the ensemble framework. This adaptability is crucial in the IoMT domain, where the landscape of network patterns and potential threats is continually shifting, necessitating an IDS that can learn and optimize in real time.

**Figure 3:**
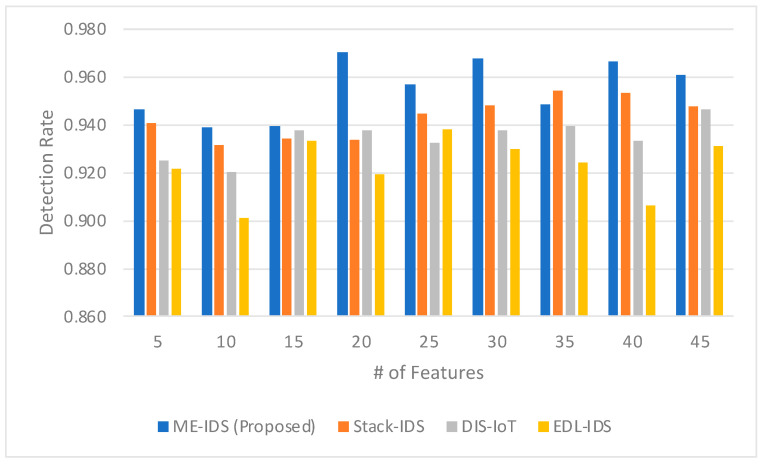
The comparison between the detection rate obtained by the proposed model and the related models.

The detection rate shown in Figure 3 presents a comprehensive overview of how the proposed ME-IDS model fares against other established IDS models, such as Stack-IDS, DIS-IoT, and EDL-IDS, across various feature set sizes. Notably, the ME-IDS model exhibits a strong detection rate, especially at the 20-feature mark, achieving a peak rate of 0.970, which significantly surpasses the corresponding rates of Stack-IDS, DIS-IoT, and EDL-IDS at 0.934, 0.938, and 0.920, respectively. This highlights the model’s ability to effectively identify intrusions when provided with a rich yet not overly complex feature set. Such a performance could be attributed to the sophisticated integration of a meta-learner within the ME-IDS model, which smartly selects and assigns weights to various classifiers, enhancing the overall detection capabilities. Additionally, the self-optimizing ensemble architecture likely contributes to maintaining high detection rates even as the number of features varies, adapting to the model’s needs and the nature of the data it processes. Although there is a slight decrease in detection rates for the ME-IDS model, with the number of features at 35 and then an increase at 40, it remains competitive, signifying the robustness of the model. In contrast, Stack-IDS shows an increase in detection rates with the number of features, peaking at 35 and 40, which suggests that Stack-IDS might require a larger feature set to perform optimally. The other two models, DIS-IoT and EDL-IDS, exhibit less variability in detection rates as the feature set size changes, but they do not reach the detection rates of ME-IDS at its peak. The data underscores the potential effectiveness of the meta-learner in optimizing detection rates within an ensemble model, especially in the context of IoMT, where the accurate and timely detection of intrusions is critical for ensuring the security and integrity of healthcare services.

The detection rate comparison in Figure 3 highlights the significant role of the meta-learner in the proposed ME-IDS model’s ability to outperform related works over a range of feature sets. The ME-IDS model’s exemplary peak detection rate at 20 features and its consistent performance with larger feature sets are indicative of the meta-learner’s effective model selection and weighting strategy. This strategy adeptly handles the complexity introduced with more features, optimizing the trade-off between detection sensitivity and the risk of overfitting. Furthermore, the self-optimizing ensemble architecture, which likely underpins the ME-IDS model, demonstrates its ability to adapt to the intrinsic data variability in IoMT environments. It achieves this by calibrating the ensemble in response to the feedback from real-world deployment, thereby sustaining high detection rates. The slight fluctuations in detection rates at higher feature counts hint at the ensemble’s dynamic adjustment capabilities, showcasing the system’s resilience in adapting to a broader feature space while maintaining competitive detection rates. This adaptability is crucial for IoMT security, where the threat landscape is constantly evolving, and the IDS must be capable of swift recalibration to maintain optimal performance.

**Figure 4:**
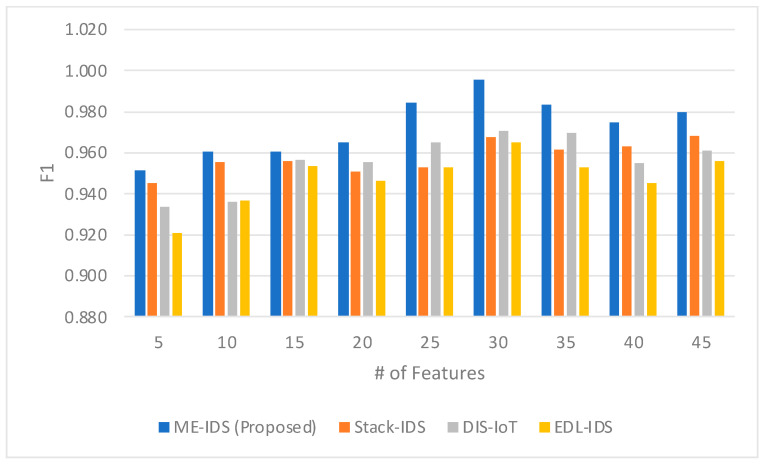
The comparison between the F1 score obtained by the proposed model and the related models.

The F1 measure comparison shown in Figure 4 between the proposed ME-IDS model and existing IDS models reveals the ME-IDS model’s superior precision and recall balance, particularly as the number of features increases. With an F1 score peaking at 0.996 for 30 features, the proposed model demonstrates an exceptional ability to maintain a high true positive rate while minimizing false positives and negatives, which is pivotal in the IoMT context, where the stakes for accurate detection are high. At 25 features, the ME-IDS model begins to notably outdistance its counterparts, achieving an F1 score of 0.985 compared to Stack-IDS, DIS-IoT, and EDL-IDS, which score 0.953, 0.965, and 0.953, respectively. This significant margin suggests that the ME-IDS model is particularly effective at integrating and utilizing a rich feature set to deliver a highly accurate intrusion detection performance. The ME-IDS’s consistently high F1 scores across the feature set spectrum showcase the nuanced capabilities of the meta-learner, which adeptly navigates the trade-off between precision and recall. In an ensemble architecture, such a meta-learner ensures that the most predictive models are given precedence, effectively synthesizing their predictions to maximize the F1 measure. This is especially evident in the performance plateau observed with higher feature counts (35 and 45), where the model maintains high F1 scores of 0.983 and 0.980, respectively, indicating the meta-learner’s proficient management of complex feature interactions. The self-optimizing nature of the ensemble likely contributes to these results, dynamically adjusting to the evolving data patterns and attack vectors characteristic of the IoMT environment. Such results underscore the effectiveness of the proposed model in delivering robust intrusion detection performance, which is crucial for the secure operation of IoMT systems.

The F1 measure comparison across the proposed ME-IDS model and other IDS models, as shown in Figure 4, provides insight into the effectiveness of the meta-learner in optimizing the ensemble architecture for the complex task of intrusion detection in the IoMT. The ME-IDS’s consistently high F1 scores, particularly the notable peak at 0.996 for 30 features, underscore the adeptness of the meta-learner in not only selecting the most appropriate features but also in weighting the individual classifiers within the ensemble to maximize both precision and recall. The meta-learner’s ability to achieve such high F1 scores indicates a sophisticated understanding of the nuanced interplay between different types of errors and their impact on the overall model performance. This balancing act is crucial in IoMT environments where false positives can be as detrimental as false negatives. Furthermore, the self-optimizing nature of the ensemble architecture, as evidenced by the sustained high F1 scores even at larger feature counts, reflects its capacity to adapt to the changing nature of the data and potential threats. The architecture’s ability to maintain performance, adapting through the meta-learner’s ongoing adjustments, suggests that it is not static but rather a dynamic system capable of evolving with the threat landscape. This is particularly pertinent in the IoMT context, where the threat vectors can change rapidly and unpredictably, necessitating an IDS that is both reactive and proactive in its learning approach. The high F1 measure achieved by the ME-IDS model thus signifies the successful application of meta-learning in conjunction with a self-optimizing ensemble architecture to produce a robust and reliable intrusion detection system for the IoMT. 

**Figure 5:**
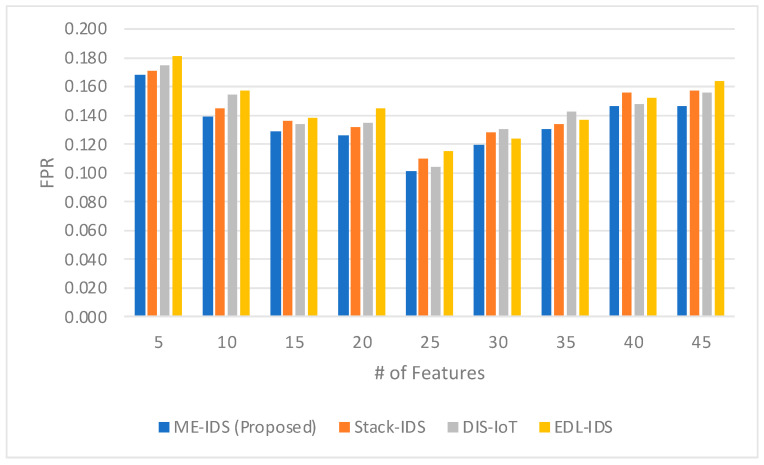
The comparison between the FPR obtained by the proposed model and the related models.

The comparison of the false positive rate (FPR) in Figure 5 measures the relationship between the proposed ME-IDS model and the related IDS works, revealing the proposed model’s proficiency in minimizing erroneous intrusion alerts. Notably, the ME-IDS model demonstrates a superior capability to reduce false alarms, which is crucial in IoMT environments where false positives can lead to unnecessary interventions or desensitization to alerts. The model showcases its lowest FPR at 25 features with a rate of 0.101, indicating a high level of specificity in identifying true threats. This outperforms the comparative models: Stack-IDS with an FPR of 0.110, DIS-IoT at 0.104, and EDL-IDS at 0.115, illustrating the ME-IDS model’s effective discrimination between normal and anomalous behaviors. This low FPR can be attributed to the fine-tuning capabilities of the meta-learner within the ME-IDS architecture, which intricately balances sensitivity and specificity. The meta-learner’s model selection and weighting functions are tailored to prioritize classifiers that not only detect intrusions with high accuracy but also with minimal false alarms. The slight increase in FPR across all models, including ME-IDS, as the number of features grows to 45 suggests a complexity threshold beyond which the specificity of the model may slightly diminish. However, the ME-IDS model’s FPR remains competitive, even at higher feature counts, indicating the self-optimizing architecture’s ability to adjust and maintain a low rate of false positives amidst an increasingly complex feature space. This emphasizes the model’s reliability and the potential to provide a trusted layer of security in the IoMT infrastructure.

The false positive rate (FPR) comparison in Figure 5 sheds light on the intricate role of the meta-learner within the ME-IDS model, especially when juxtaposed with related works. The ME-IDS model’s capacity to sustain lower FPRs across varying numbers of features is a testament to the meta-learner’s adept selection and weighting of classifiers. This is evident in the notably lower FPR at 25 features, where the proposed model achieves a rate of 0.101, significantly outperforming other models. Such an achievement suggests that the meta-learner is effectively distinguishing between noise and true signal, a capability that is particularly beneficial in the IoMT context, where false alarms can be costly and disruptive. Moreover, the meta-learner’s strategy, which likely involves a nuanced understanding of the IoMT data patterns, enables the model to maintain a competitive FPR even as the feature space becomes more complex, as indicated by the FPRs observed with 40 and 45 features. The self-optimizing architecture of the ensemble further enhances this performance, adjusting to new data and potential threats dynamically. It implies an underlying mechanism that can recalibrate the ensemble’s decision threshold in response to real-time feedback, thus preserving a low FPR. This adaptability is crucial, as the IoMT environment is not only diverse but also evolves rapidly, with new device types and usage patterns continually emerging. The consistently low FPR of the ME-IDS model underlines the successful integration of the meta-learner with the self-optimizing ensemble architecture, culminating in a robust IDS that minimizes the rate of false positives without compromising the detection capabilities, thereby upholding the integrity and trustworthiness of IoMT security systems.

With respect to resource consumption, Table 1 shows that the proposed ME-IDS model exhibits better resource utilization compared to other models due to its efficient design. The model demonstrates decreased CPU utilization (ranging from 41% to 57% during training and 20% to 30% during inference) and memory consumption (occupying 45% to 49% of RAM). Additionally, it exhibits a notable reduction in inference time, processing each input batch in 0.02 to 0.05 s. The enhancements can be attributed to various design elements like dynamic weighting and meta-learning, which optimize the distribution of computational resources and prioritize the most efficient classifiers. Likewise, regularization techniques such as dropout and L2 regularization prevent overfitting and efficiently manage memory usage. Similarly, model pruning simplifies the ensemble by eliminating less effective classifiers. By incorporating these techniques, the proposed ME-IDS model not only enhances the precision and flexibility of detection but also guarantees better resource utilization, rendering it suitable for resource-constrained environments like IoMT.

Although the proposed system shows enhanced performance in various aspects, it is crucial to consider and tackle the possibility of unexplored threat vectors or attack types. The heterogeneous composition of the ensemble IDS is a crucial determinant in the detection of novel and unrecognized security risks. The system utilizes a combination of multiple detection techniques in the ensemble, which allows it to capitalize on the advantages of different classifiers, thereby improving its capability to identify new attacks. Every classifier within the ensemble could focus on specific aspects of threat detection, guaranteeing thorough coverage and minimizing the chances of overlooking novel attack patterns. The ensemble’s integration of anomaly detection techniques in conjunction with signature-based detection is highly effective in identifying atypical behavior that may indicate novel or unfamiliar attacks. This dual methodology guarantees that the system can identify both known and unknown threats.

The integration of the IDS into existing IoMT environments poses significant challenges, especially in terms of compatibility with legacy systems and technologies. To tackle these problems, we suggest implementing a modular structure that permits adaptable integration with different components of current IoMT frameworks. This will enable a gradual implementation process to minimize any disruptions. Following common interoperability standards like HL7, DICOM, and IEEE 11073 guarantees smooth communication with older systems and a variety of medical devices. The integration layer of the IDS can be tailored to meet the specific needs of various IoMT environments. It manages tasks such as data transformation, protocol conversion, and other necessary modifications. By implementing the IDS in phases, it is possible to gradually adapt to its use. This approach minimizes the risk of compatibility issues by first deploying the system in a controlled environment and then gradually expanding its scope. Thorough testing and validation, which includes rigorous compatibility testing with different older technologies and conducting pilot studies, are performed to guarantee that the IDS functions smoothly with current systems. By implementing these tactics, the proposed IDS can be seamlessly incorporated into current IoMT frameworks, guaranteeing harmonious coexistence with outdated systems and technologies while simultaneously bolstering the overall security and functionality of the IoMT ecosystem.

## 5. Conclusions

The study explores the integration of ensemble learning techniques, specifically meta-learning, for improving intrusion detection systems (IDS) in the Internet of Medical Things (IoMT) environment. The research demonstrates that an ensemble-based IDS, optimized with meta-learning strategies, can significantly improve the detection of cyber threats in IoMT networks. The proposed performance-driven weighted meta-learning technique dynamically assigns voting weights to classifiers based on their accuracy, loss metrics, and confidence levels. The experimental evaluation reveals the model’s superiority in accurately detecting intrusions and demonstrates remarkable adaptability and efficiency in handling complex IoMT data streams. The research emphasizes the importance of advanced security measures to safeguard sensitive medical data and ensure healthcare system reliability amid the proliferation of connected devices. Future research should focus on refining meta-learning algorithms and exploring their applicability in real-world IoMT scenarios. The integration of meta-learning in ensemble-based IDS holds significant promise for advancing cybersecurity measures and ensuring the integrity, confidentiality, and availability of critical healthcare data in connected environments.

## Figures and Tables

**Table 1:** Comparison of resource consumption between the proposed and related works.

Performance Measure	ME-IDS (Proposed)	Stack-IDS	DIS-IoT	EDL-IDS
CPU Usage	41–57% during peak training; 20–30% during inference	44–61% during peak training; 35–42% during inference	43–55% during peak training; 37–48% during inference	50–72% during peak training; 45–62% during inference
Memory Usage	45–49% of RAM	47–51% of RAM	49–55% of RAM	49–65% of RAM
Inference Time	0.02–0.05 s per input batch	0.11–0.14 s per input batch	0.17–0.2 s per input batch	0.14–0.23 s per input batch

## Data Availability

Data are contained within the article.
